# Supplementary material for: High Throughput Preparation of Ag-Zn Alloy Thin Films for the Electrocatalytic Reduction of CO2 to CO
Source: Materials (Basel). 2022 Oct 4;15(19):6892. doi: 10.3390/ma15196892 (PMC9571298; doi:10.3390/ma15196892)
Supplement: Supplementary file 1 [file materials-15-06892-s001.zip › materials-1925096-supplementary.pdf]

Supplementary Materials

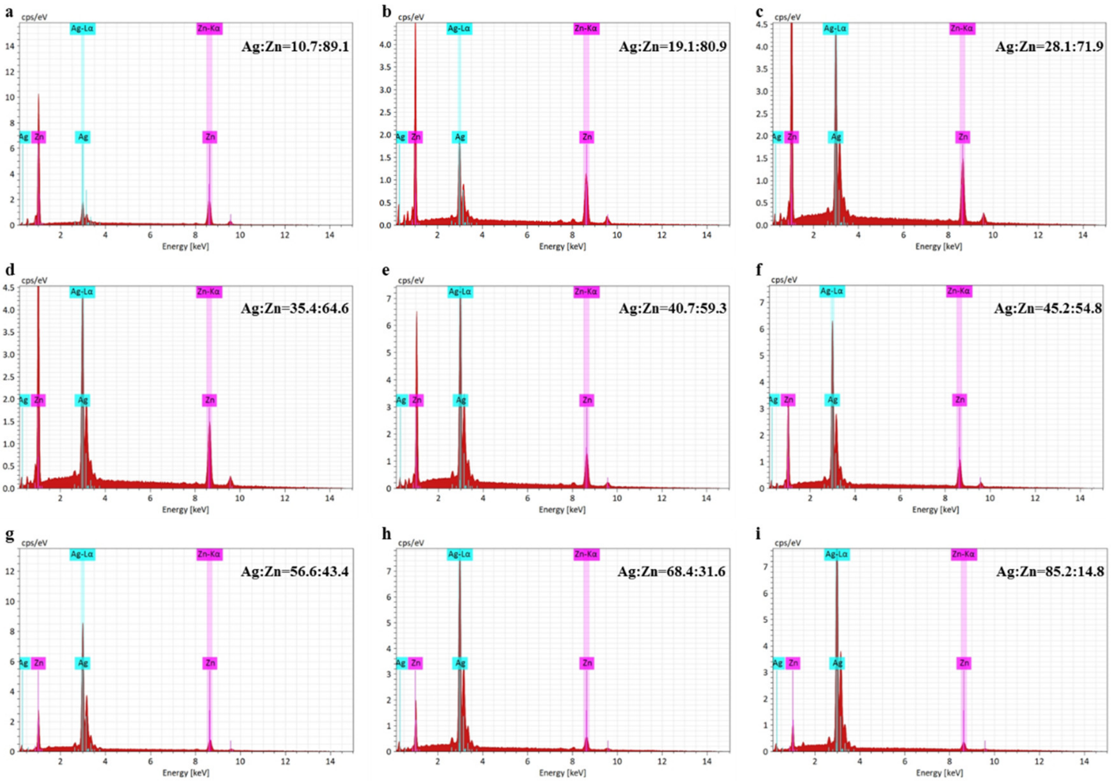

Figure S1. Typical EDX spectra of (**a**) $Ag_{10}Zn_{90}$, (**b**) $Ag_{20}Zn_{80}$, (c) $Ag_{30}Zn_{70}$, (**d**) $Ag_{35}Zn_{65}$, (**e**) $Ag_{40}Zn_{60}$, (**f**) $Ag_{45}Zn_{55}$, (**g**) $Ag_{55}Zn_{45}$, (**h**) $Ag_{70}Zn_{30}$ and (**i**) $Ag_{85}Zn_{15}$ films .

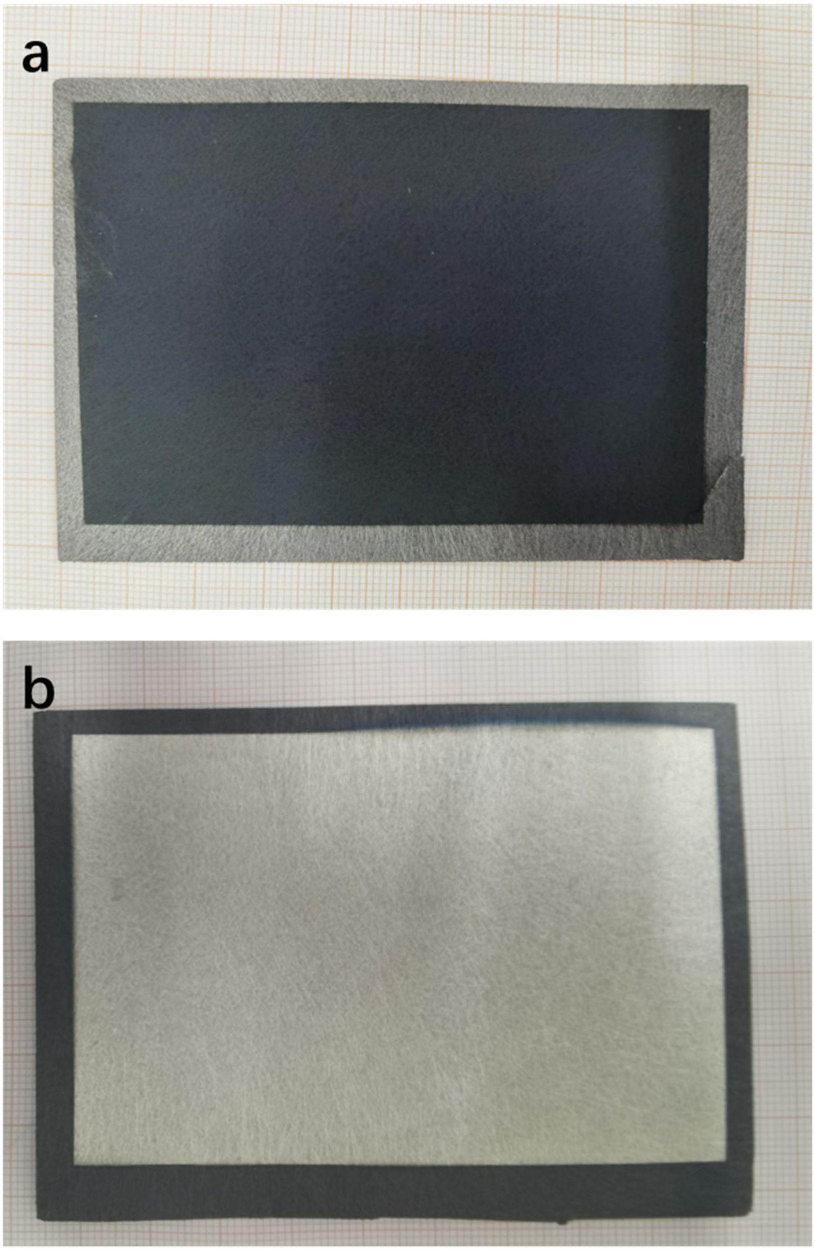

Figure S2. Macroscopic view of the (**a**) Zn film and (**b**) Ag film.

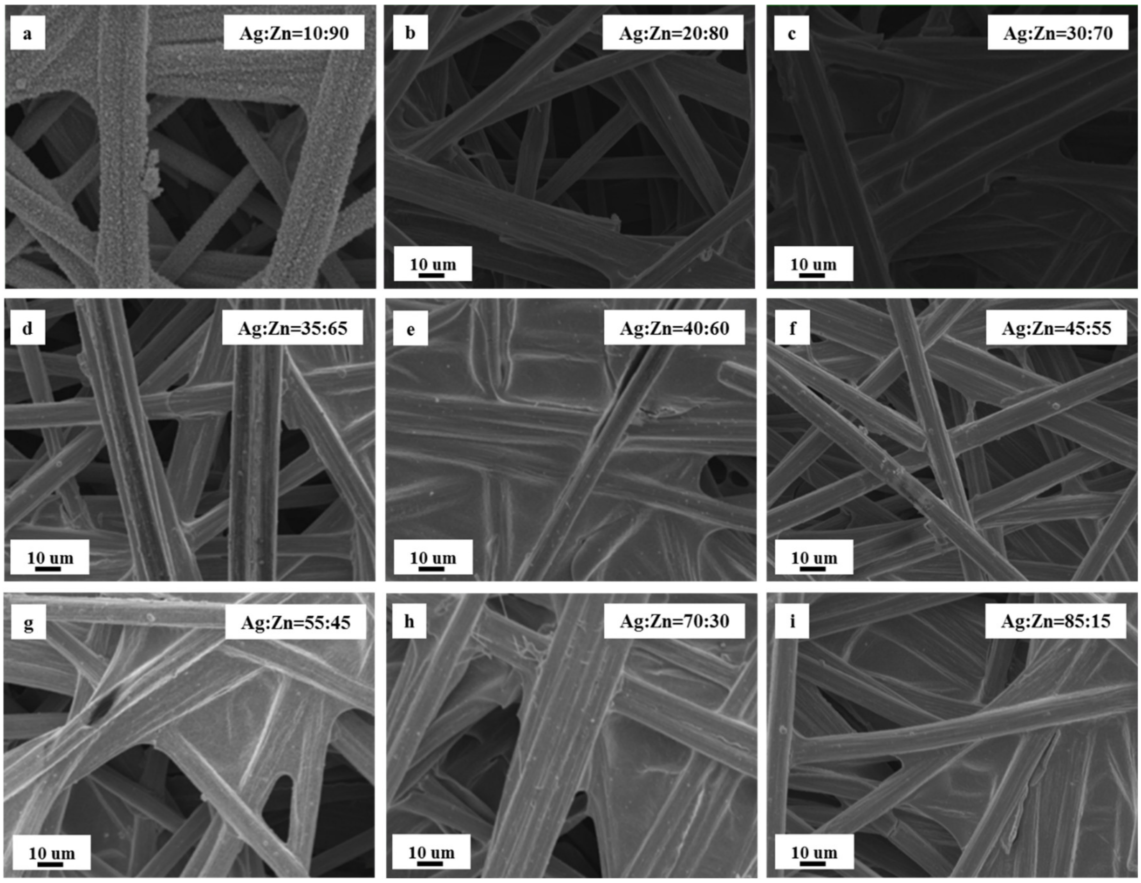

Figure S3. SEM images of (**a**) $Ag_{10}Zn_{90}$, (**b**) $Ag_{20}Zn_{80}$, (**c**) $Ag_{30}Zn_{70}$, (**d**) $Ag_{35}Zn_{65}$, (**e**) $Ag_{40}Zn_{60}$, (**f**) $Ag_{45}Zn_{55}$, (**g**) $Ag_{55}Zn_{45}$, (**h**) $Ag_{70}Zn_{30}$ and (**i**) $Ag_{85}Zn_{15}$ films.

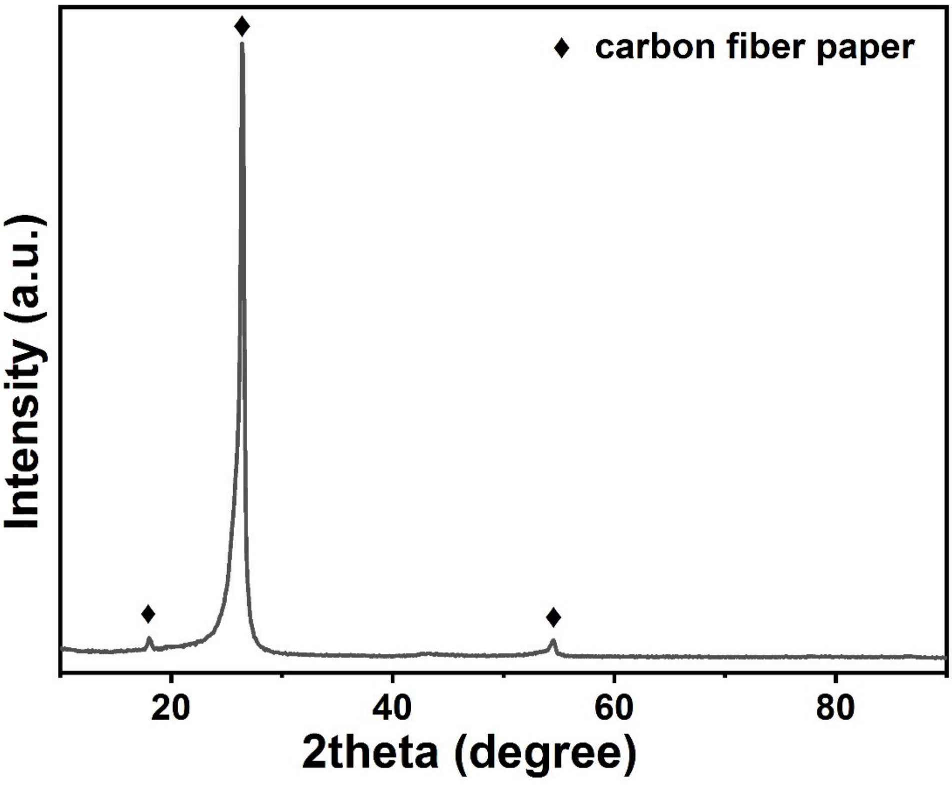

Figure S4. XRD pattern of the CFP.

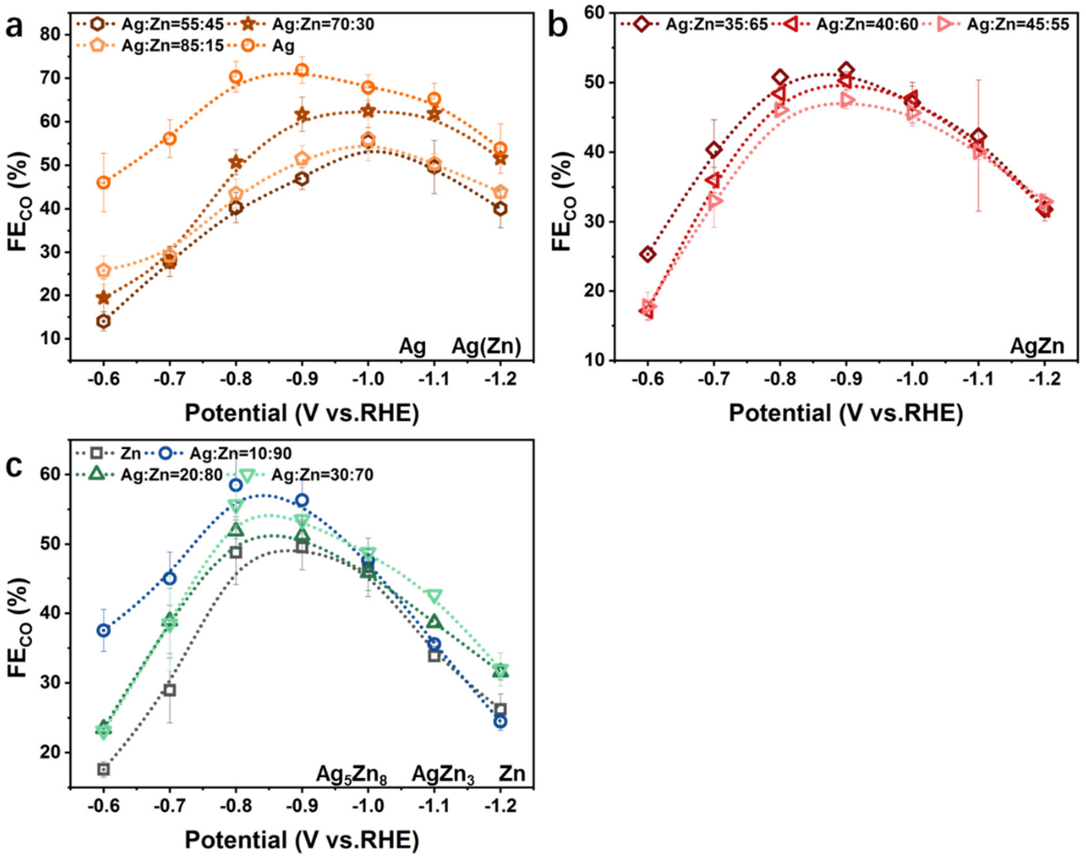

Figure S5. FE_{CO} for the (**a**) Ag, Ag(Zn), (**b**) AgZn, (**c**) Ag_5Zn_8, $AgZn_3$, and Zn phase catalysts.

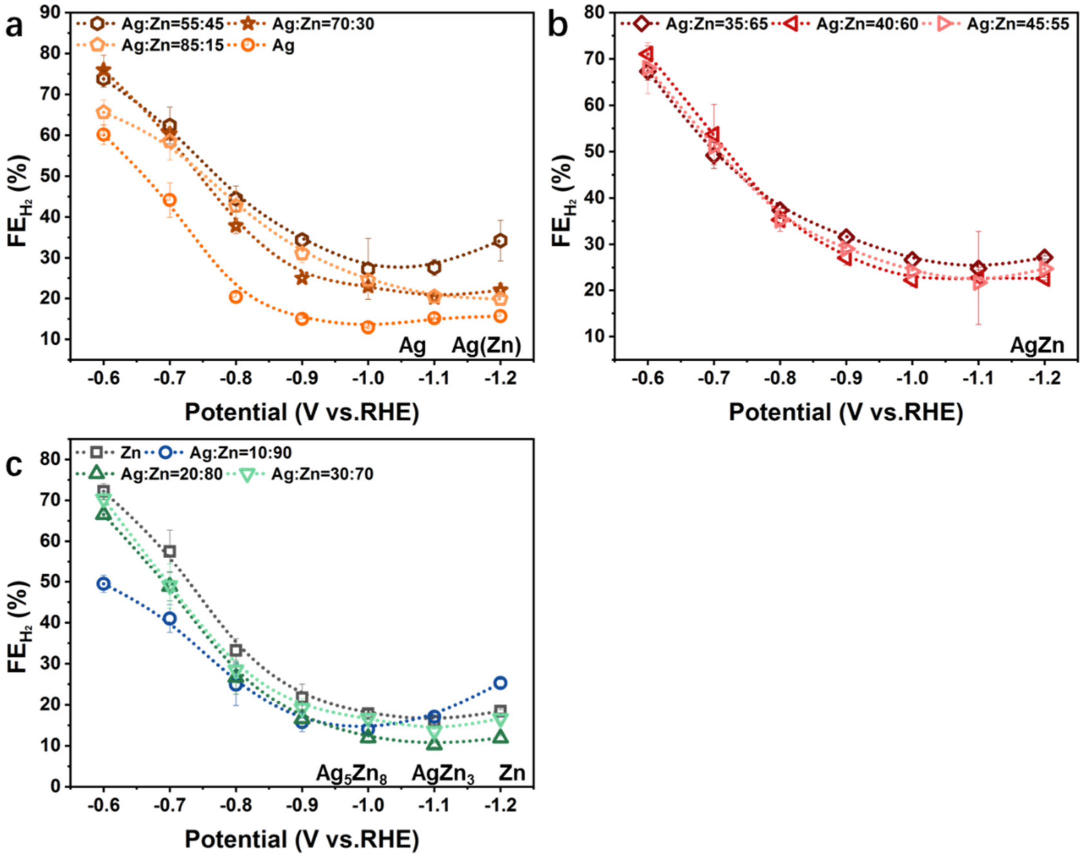

Figure S6. FE_{H_2} for the (**a**) Ag, Ag(Zn), (**b**) AgZn, (**c**) Ag_5Zn_8, $AgZn_3$, and Zn phase catalysts.

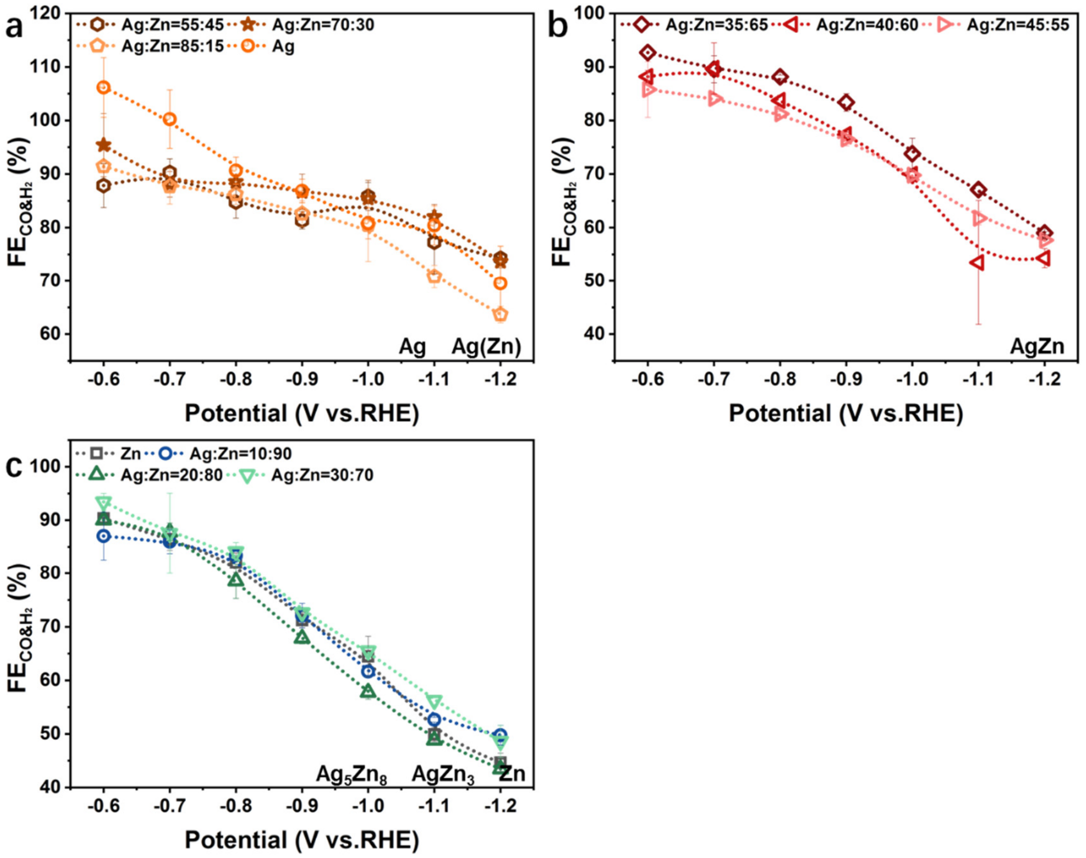

Figure S7. FE$_{CO\&H_2}$ for the (**a**) Ag, Ag(Zn), (**b**) AgZn, (**c**) Ag_5Zn_8, $AgZn_3$, and Zn phase catalysts.

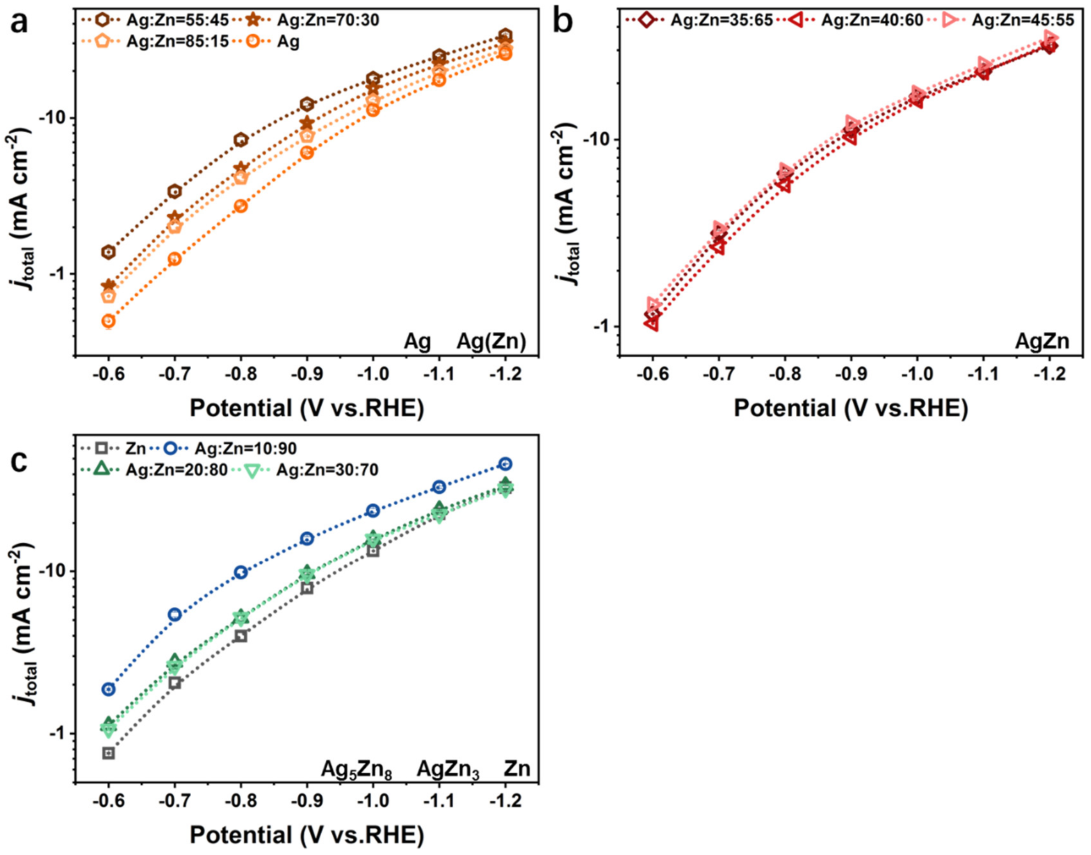

Figure S8. j_{total} for the (**a**) Ag, Ag(Zn), (**b**) AgZn, (**c**) Ag_5Zn_8, $AgZn_3$, and Zn phase catalysts.

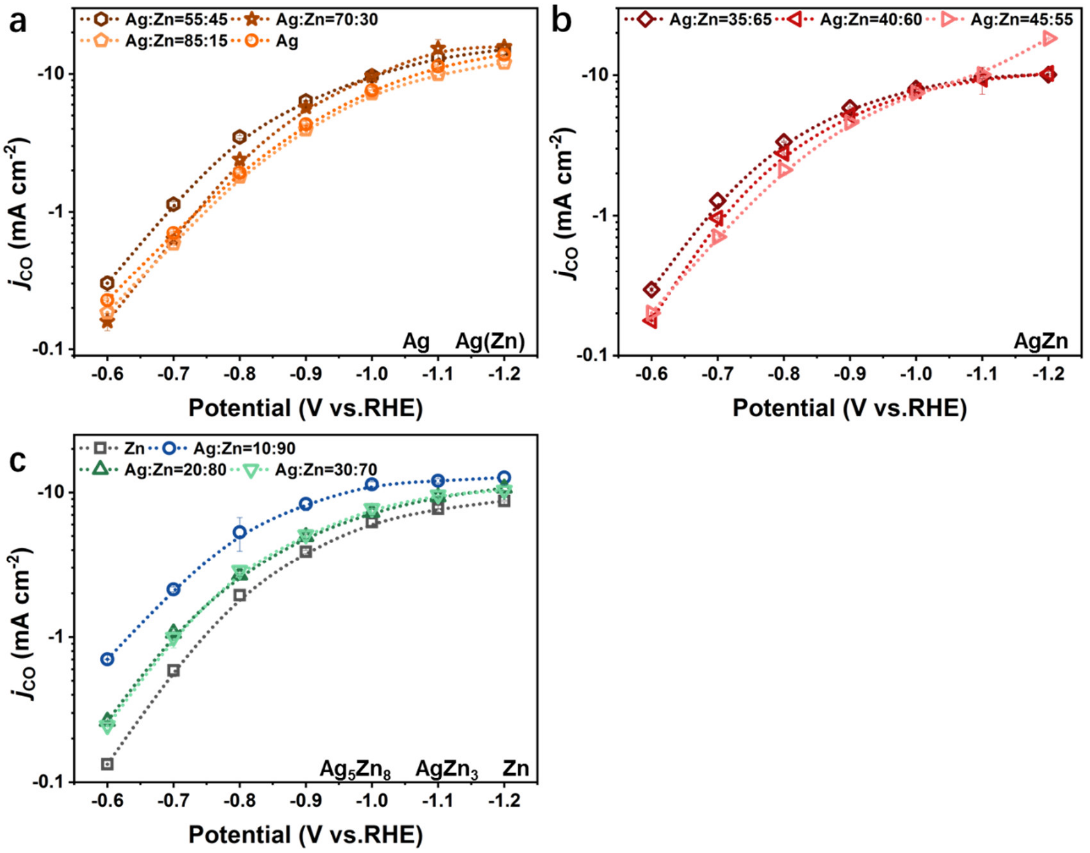

Figure S9. j_{CO} for the (**a**) Ag, Ag(Zn), (**b**) AgZn, (**c**) Ag_5Zn_8, $AgZn_3$, and Zn phase catalysts.

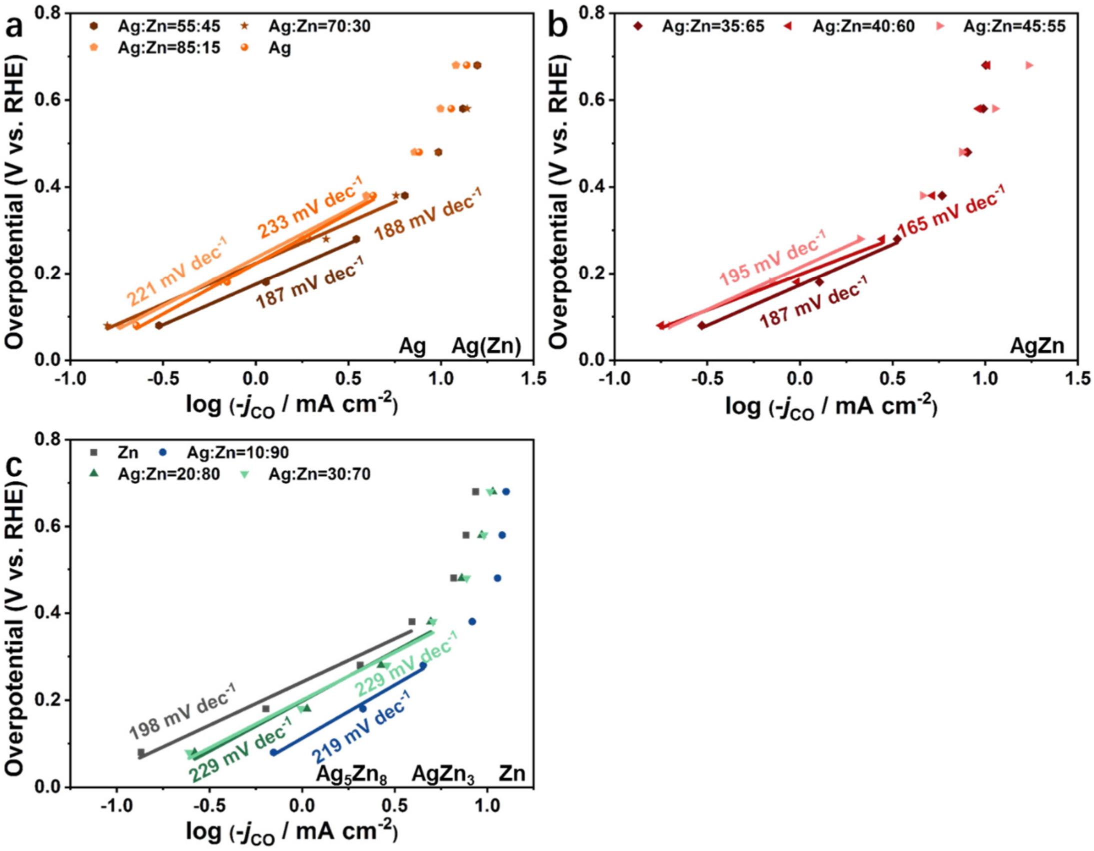

Figure S10. Tafel plots of the (**a**) Ag, Ag(Zn), (**b**) AgZn, (**c**) Ag_5Zn_8, $AgZn_3$ and Zn phase catalysts.

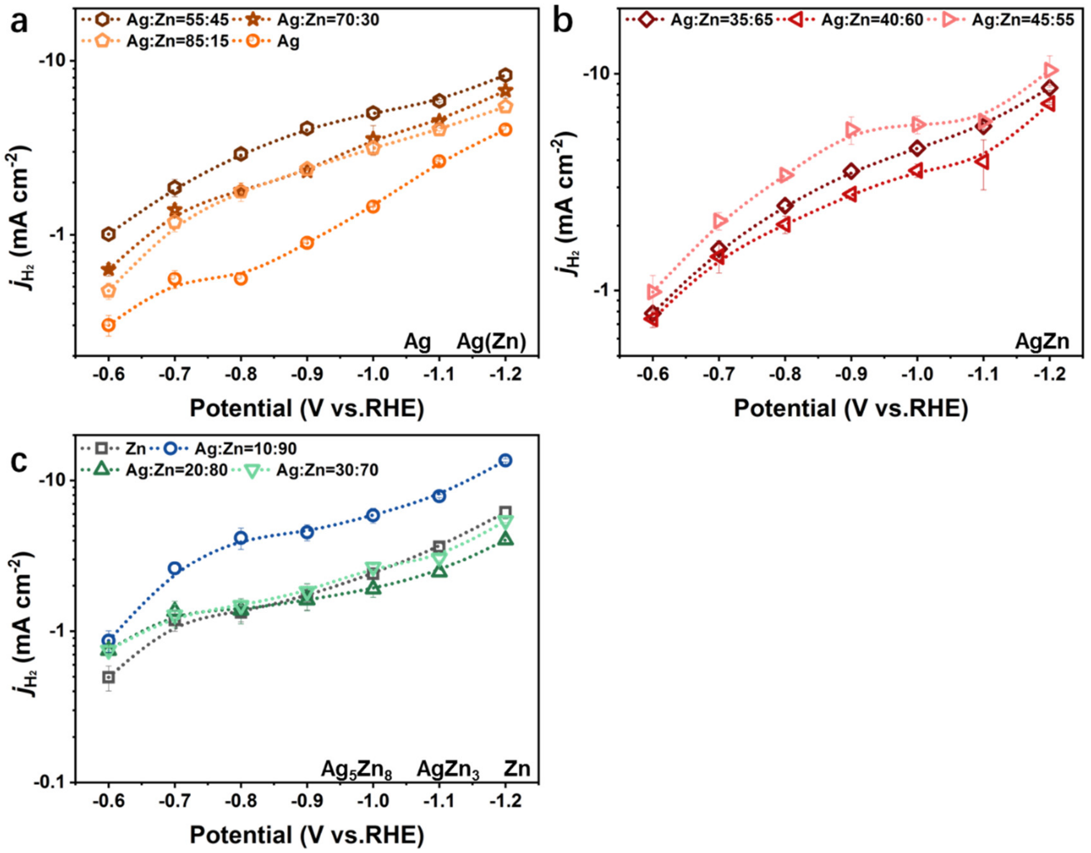

Figure S11. j_{H_2} for the (**a**) Ag, Ag(Zn), (**b**) AgZn, (**c**) Ag_5Zn_8, $AgZn_3$, and Zn phase catalysts.
